# (μ-Naphthalene-1,5-disulfonato-κ^2^ *O* ^1^:*O* ^5^)bis­[triaqua­(glycinato-κ^2^ *N*,*O*)copper(II)]

**DOI:** 10.1107/S1600536812019332

**Published:** 2012-05-05

**Authors:** Shan Gao, Seik Weng Ng

**Affiliations:** a Key Laboratory of Functional Inorganic Material Chemistry, Ministry of Education, Heilongjiang University, Harbin 150080, People’s Republic of China; b Department of Chemistry, University of Malaya, 50603 Kuala Lumpur, Malaysia; c Chemistry Department, Faculty of Science, King Abdulaziz University, PO Box 80203 Jeddah, Saudi Arabia

## Abstract

In the title compound, [Cu_2_(C_2_H_4_NO_2_)_2_(C_10_H_6_O_6_S_2_)(H_2_O)_6_], the naphthalene­disulfonate group lies on a center of inversion and bridges two glycinate-chelated Cu^II^ atoms. The Cu^II^ atom exists in a CuNO_4_ square-pyramidal geometry that is distorted towards an octa­hedron owing to a long Cu—O_sulfonate_ bond [2.636 (2) Å]. In the crystal, extensive N—H⋯O and O—H⋯O hydrogen bonds link adjacent mol­ecules into a three-dimensional network

## Related literature

For a review of metal arene­sulfonates, see: Cai (2004 ▶).
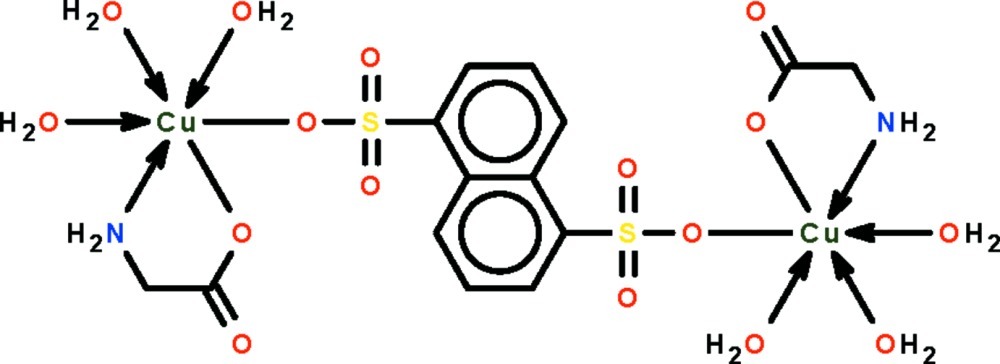


## Experimental

### 

#### Crystal data

[Cu_2_(C_2_H_4_NO_2_)_2_(C_10_H_6_O_6_S_2_)(H_2_O)_6_]
*M* *_r_* = 669.57
Monoclinic, 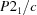

*a* = 5.802 (3) Å
*b* = 11.341 (6) Å
*c* = 17.613 (8) Å
β = 99.793 (18)°
*V* = 1142.1 (9) Å^3^
*Z* = 2
Mo *K*α radiation
μ = 2.13 mm^−1^
*T* = 293 K
0.38 × 0.26 × 0.19 mm

#### Data collection

Rigaku R-AXIS RAPID IP diffractometer
Absorption correction: multi-scan (*ABSCOR*; Higashi, 1995 ▶) *T*_min_ = 0.498, *T*_max_ = 0.688
10939 measured reflections
2615 independent reflections
2482 reflections with *I* > 2σ(*I*)
*R*_int_ = 0.022

#### Refinement

*R*[*F* ^2^ > 2σ(*F* ^2^)] = 0.026
*wR*(*F* ^2^) = 0.073
*S* = 1.07
2615 reflections
166 parameters
H-atom parameters constrained
Δρ_max_ = 0.47 e Å^−3^
Δρ_min_ = −0.63 e Å^−3^

### 

Data collection: *RAPID-AUTO* (Rigaku, 1998 ▶); cell refinement: *RAPID-AUTO*; data reduction: *CrystalClear* (Rigaku/MSC, 2002 ▶); program(s) used to solve structure: *SHELXS97* (Sheldrick, 2008 ▶); program(s) used to refine structure: *SHELXL97* (Sheldrick, 2008 ▶); molecular graphics: *X-SEED* (Barbour, 2001 ▶); software used to prepare material for publication: *publCIF* (Westrip, 2010 ▶).

## Supplementary Material

Crystal structure: contains datablock(s) global, I. DOI: 10.1107/S1600536812019332/xu5523sup1.cif

Structure factors: contains datablock(s) I. DOI: 10.1107/S1600536812019332/xu5523Isup2.hkl

Additional supplementary materials:  crystallographic information; 3D view; checkCIF report

## Figures and Tables

**Table 1:** Hydrogen-bond geometry (Å, °)

*D*—H⋯*A*	*D*—H	H⋯*A*	*D*⋯*A*	*D*—H⋯*A*
O1*w*—H11⋯O2^i^	0.84	2.02	2.741 (2)	143
O1*w*—H12⋯O5^ii^	0.84	2.10	2.785 (2)	139
O2*w*—H21⋯O4^iii^	0.84	2.00	2.773 (2)	152
O2*w*—H22⋯O4^iv^	0.84	2.02	2.823 (2)	158
O3*w*—H31⋯O2^ii^	0.84	1.91	2.676 (2)	151
O3*w*—H32⋯O3^iv^	0.84	1.93	2.691 (2)	150
N1—H1⋯O5	0.88	2.53	3.079 (3)	121
N1—H2⋯O2^ii^	0.88	2.50	3.194 (3)	137

Symmetry codes: (i) 
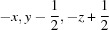
; (ii) 
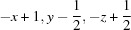
; (iii) 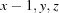; (iv) 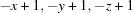.

**Fig. 1.**
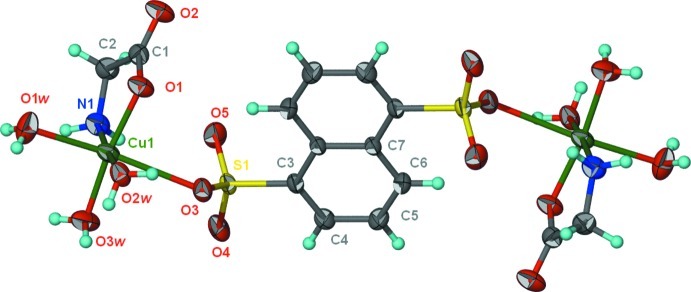
Thermal ellipsoid plot (Barbour, 2001) of the title compound at the 70% probability level; hydrogen atoms are drawn as spheres of arbitrary radius. Symmetry-related atoms are not labeled.

## supplementary crystallographic information

### Comment

Metal arenesulfonates are generally crystalline compounds; in some, the metal is connected to the arenesulfonate by a covalent bond whereas in others, the arenesulfonate interacts indirectly with the metal center in an outer-sphere type of coordination (Cai, 2004). In the title compound (Scheme I), the Cu^II^ atom exists in a CuNO_4_ square-pyramidal geometry that is distorted towards an octahedron owing to the long Cu–O_sulfonate_ bond (2.636 (2) Å). The atom lies above the square plane (r.m.s. deviation 0.082 Å) and the apical water molecule lies 2.371 (2) Å above the plane (Fig.1). Extensive N–H···O and O–H···O hydrogen bonds link adjacent molecules into a three-dimensional network (Table 1).

### Experimental

Dicopper carbonate dihydroxide (1 mmol, 221 mg), glycine (2 mmol, 150 mg) 1,5-naphthalenedisulfonic acid tetrahydrate (2 mmol, 720 mg) were dissolved in water (10 ml). The solution was heated for 5 h and then filtered. Blue crystals separated from the solution after several days.

### Refinement

H-atoms were placed in calculated positions (C–H 0.93–0.97, N–H 0.88, O–H 0.84 Å) and were included in the refinement in the riding model approximation, with *U*(H) set to 1.2–1.5*U*(*C*,*N*,*O*).

The (2 2 3) reflection was omitted owing to bad disagreement.

### Crystal data

**Table:**

[Cu_2_(C_2_H_4_NO_2_)_2_(C_10_H_6_O_6_S_2_)(H_2_O)_6_]	*F*(000) = 684
*M**_r_* = 669.57	*D*_x_ = 1.947 Mg m^−^^3^
Monoclinic, *P*2_1_/*c*	Mo *K*α radiation, λ = 0.71073 Å
Hall symbol: -P 2ybc	Cell parameters from 10177 reflections
*a* = 5.802 (3) Å	θ = 3.6–27.5°
*b* = 11.341 (6) Å	µ = 2.13 mm^−^^1^
*c* = 17.613 (8) Å	*T* = 293 K
β = 99.793 (18)°	Prism, blue
*V* = 1142.1 (9) Å^3^	0.38 × 0.26 × 0.19 mm
*Z* = 2	

### Data collection

**Table:**

Rigaku R-AXIS RAPID IP diffractometer	2615 independent reflections
Radiation source: fine-focus sealed tube	2482 reflections with *I* > 2σ(*I*)
Graphite monochromator	*R*_int_ = 0.022
ω scan	θ_max_ = 27.5°, θ_min_ = 3.6°
Absorption correction: multi-scan (*ABSCOR*; Higashi, 1995)	*h* = −7→6
*T*_min_ = 0.498, *T*_max_ = 0.688	*k* = −14→14
10939 measured reflections	*l* = −21→22

### Refinement

**Table:**

Refinement on *F*^2^	Primary atom site location: structure-invariant direct methods
Least-squares matrix: full	Secondary atom site location: difference Fourier map
*R*[*F*^2^ > 2σ(*F*^2^)] = 0.026	Hydrogen site location: inferred from neighbouring sites
*wR*(*F*^2^) = 0.073	H-atom parameters constrained
*S* = 1.07	*w* = 1/[σ^2^(*F*_o_^2^) + (0.0429*P*)^2^ + 0.6921*P*] where *P* = (*F*_o_^2^ + 2*F*_c_^2^)/3
2615 reflections	(Δ/σ)_max_ = 0.001
166 parameters	Δρ_max_ = 0.47 e Å^−^^3^
0 restraints	Δρ_min_ = −0.63 e Å^−^^3^

### Fractional atomic coordinates and isotropic or equivalent isotropic displacement parameters (Å^2^)

**Table:**

	*x*	*y*	*z*	*U*_iso_*/*U*_eq_	
Cu1	0.35965 (4)	0.51409 (2)	0.341914 (12)	0.02354 (9)	
S1	0.70836 (8)	0.73372 (4)	0.44741 (2)	0.02159 (11)	
O1	0.1995 (2)	0.64747 (12)	0.28644 (8)	0.0287 (3)	
O2	0.2226 (3)	0.77425 (13)	0.19250 (8)	0.0343 (3)	
O1w	0.1638 (3)	0.37202 (15)	0.26192 (9)	0.0397 (4)	
H11	0.0956	0.3256	0.2879	0.060*	
H12	0.2599	0.3342	0.2407	0.060*	
O2w	0.1040 (3)	0.50030 (11)	0.40277 (8)	0.0265 (3)	
H21	0.0939	0.5633	0.4271	0.040*	
H22	0.1325	0.4441	0.4340	0.040*	
O3w	0.5629 (3)	0.39956 (14)	0.40378 (8)	0.0369 (3)	
H31	0.5917	0.3436	0.3756	0.055*	
H32	0.4956	0.3734	0.4389	0.055*	
O3	0.4994 (2)	0.66501 (11)	0.45410 (7)	0.0270 (3)	
O4	0.9218 (3)	0.67519 (12)	0.48533 (8)	0.0327 (3)	
O5	0.7143 (3)	0.76531 (13)	0.36808 (8)	0.0345 (3)	
N1	0.5974 (3)	0.53828 (15)	0.27352 (9)	0.0280 (3)	
H1	0.7251	0.5697	0.2999	0.034*	
H2	0.6344	0.4702	0.2548	0.034*	
C1	0.2931 (3)	0.68561 (16)	0.23169 (10)	0.0236 (3)	
C2	0.4970 (3)	0.61731 (17)	0.21047 (11)	0.0279 (4)	
H2A	0.4444	0.5714	0.1643	0.033*	
H2B	0.6160	0.6719	0.1996	0.033*	
C3	0.6918 (3)	0.86710 (14)	0.49908 (9)	0.0200 (3)	
C4	0.8728 (3)	0.89338 (16)	0.55662 (10)	0.0258 (4)	
H4	0.9958	0.8404	0.5691	0.031*	
C5	0.8744 (4)	1.00050 (17)	0.59723 (12)	0.0275 (4)	
H5	0.9982	1.0174	0.6366	0.033*	
C6	0.6966 (3)	1.07958 (16)	0.57946 (10)	0.0239 (3)	
H6	0.7024	1.1507	0.6060	0.029*	
C7	0.5021 (3)	1.05448 (14)	0.52074 (9)	0.0186 (3)	

### Atomic displacement parameters (Å^2^)

**Table:**

	*U*^11^	*U*^22^	*U*^33^	*U*^12^	*U*^13^	*U*^23^
Cu1	0.02546 (13)	0.02263 (14)	0.02478 (14)	0.00560 (8)	0.01067 (9)	0.00671 (8)
S1	0.0305 (2)	0.01428 (19)	0.0221 (2)	0.00179 (15)	0.01062 (16)	−0.00129 (14)
O1	0.0311 (6)	0.0273 (7)	0.0307 (7)	0.0076 (5)	0.0134 (5)	0.0098 (5)
O2	0.0379 (7)	0.0294 (7)	0.0378 (8)	0.0054 (6)	0.0131 (6)	0.0152 (6)
O1w	0.0462 (9)	0.0409 (9)	0.0350 (8)	−0.0102 (7)	0.0153 (7)	−0.0150 (7)
O2w	0.0324 (7)	0.0199 (6)	0.0310 (7)	0.0022 (5)	0.0162 (6)	0.0001 (5)
O3w	0.0491 (8)	0.0386 (8)	0.0266 (7)	0.0221 (7)	0.0162 (6)	0.0093 (6)
O3	0.0362 (7)	0.0196 (6)	0.0270 (6)	−0.0049 (5)	0.0107 (5)	−0.0012 (5)
O4	0.0352 (7)	0.0224 (6)	0.0420 (8)	0.0107 (6)	0.0106 (6)	−0.0027 (6)
O5	0.0529 (9)	0.0306 (7)	0.0241 (6)	−0.0039 (6)	0.0187 (6)	−0.0018 (5)
N1	0.0273 (8)	0.0283 (8)	0.0302 (8)	0.0054 (6)	0.0103 (6)	0.0031 (6)
C1	0.0254 (8)	0.0210 (8)	0.0246 (8)	−0.0019 (7)	0.0046 (7)	0.0005 (6)
C2	0.0325 (9)	0.0286 (9)	0.0247 (8)	0.0019 (7)	0.0113 (7)	0.0021 (7)
C3	0.0266 (8)	0.0145 (7)	0.0202 (7)	0.0013 (6)	0.0076 (6)	−0.0006 (6)
C4	0.0259 (8)	0.0224 (8)	0.0282 (9)	0.0057 (7)	0.0021 (7)	0.0003 (7)
C5	0.0274 (9)	0.0269 (9)	0.0255 (9)	−0.0008 (7)	−0.0033 (7)	−0.0042 (7)
C6	0.0288 (8)	0.0194 (8)	0.0226 (8)	−0.0018 (7)	0.0023 (7)	−0.0037 (6)
C7	0.0239 (8)	0.0142 (7)	0.0183 (7)	0.0001 (6)	0.0051 (6)	0.0000 (6)

### Geometric parameters (Å, º)

**Table:**

Cu1—O1	1.9485 (15)	O3w—H32	0.8400
Cu1—O3w	1.9566 (15)	N1—C2	1.468 (2)
Cu1—O2w	1.9783 (16)	N1—H1	0.8800
Cu1—N1	1.9988 (18)	N1—H2	0.8800
Cu1—O1w	2.3081 (17)	C1—C2	1.513 (3)
Cu1—O3	2.636 (2)	C2—H2A	0.9700
S1—O5	1.4486 (15)	C2—H2B	0.9700
S1—O4	1.4626 (15)	C3—C4	1.363 (3)
S1—O3	1.4629 (15)	C3—C7^i^	1.430 (2)
S1—C3	1.7763 (18)	C4—C5	1.409 (3)
O1—C1	1.261 (2)	C4—H4	0.9300
O2—C1	1.248 (2)	C5—C6	1.362 (3)
O1w—H11	0.8400	C5—H5	0.9300
O1w—H12	0.8400	C6—C7	1.424 (2)
O2w—H21	0.8400	C6—H6	0.9300
O2w—H22	0.8400	C7—C3^i^	1.430 (2)
O3w—H31	0.8400	C7—C7^i^	1.434 (3)
			
O1—Cu1—O3w	170.20 (7)	Cu1—N1—H1	110.0
O1—Cu1—O2w	89.75 (6)	C2—N1—H2	110.0
O3w—Cu1—O2w	94.72 (7)	Cu1—N1—H2	110.0
O1—Cu1—N1	84.86 (6)	H1—N1—H2	108.4
O3w—Cu1—N1	90.81 (7)	O2—C1—O1	123.76 (17)
O2w—Cu1—N1	174.45 (6)	O2—C1—C2	118.17 (16)
O1—Cu1—O1w	95.31 (7)	O1—C1—C2	118.03 (16)
O3w—Cu1—O1w	93.67 (8)	N1—C2—C1	110.61 (15)
O2w—Cu1—O1w	86.50 (6)	N1—C2—H2A	109.5
N1—Cu1—O1w	92.67 (7)	C1—C2—H2A	109.5
O5—S1—O4	113.29 (9)	N1—C2—H2B	109.5
O5—S1—O3	111.43 (9)	C1—C2—H2B	109.5
O4—S1—O3	111.76 (9)	H2A—C2—H2B	108.1
O5—S1—C3	107.21 (9)	C4—C3—C7^i^	121.37 (15)
O4—S1—C3	105.54 (8)	C4—C3—S1	117.72 (13)
O3—S1—C3	107.12 (8)	C7^i^—C3—S1	120.89 (13)
C1—O1—Cu1	114.77 (12)	C3—C4—C5	120.24 (16)
Cu1—O1w—H11	109.5	C3—C4—H4	119.9
Cu1—O1w—H12	109.5	C5—C4—H4	119.9
H11—O1w—H12	109.5	C6—C5—C4	120.77 (17)
Cu1—O2w—H21	109.5	C6—C5—H5	119.6
Cu1—O2w—H22	109.5	C4—C5—H5	119.6
H21—O2w—H22	109.5	C5—C6—C7	120.72 (17)
Cu1—O3w—H31	109.5	C5—C6—H6	119.6
Cu1—O3w—H32	109.5	C7—C6—H6	119.6
H31—O3w—H32	109.5	C6—C7—C3^i^	123.13 (15)
C2—N1—Cu1	108.28 (12)	C6—C7—C7^i^	119.03 (19)
C2—N1—H1	110.0	C3^i^—C7—C7^i^	117.83 (18)
			
O2w—Cu1—O1—C1	−174.25 (13)	O4—S1—C3—C4	4.06 (16)
N1—Cu1—O1—C1	4.42 (13)	O3—S1—C3—C4	123.29 (15)
O1w—Cu1—O1—C1	−87.80 (14)	O5—S1—C3—C7^i^	61.24 (16)
O1—Cu1—N1—C2	−13.65 (13)	O4—S1—C3—C7^i^	−177.71 (14)
O3w—Cu1—N1—C2	175.17 (13)	O3—S1—C3—C7^i^	−58.48 (16)
O1w—Cu1—N1—C2	81.45 (13)	C7^i^—C3—C4—C5	−1.1 (3)
Cu1—O1—C1—O2	−175.95 (15)	S1—C3—C4—C5	177.09 (15)
Cu1—O1—C1—C2	6.4 (2)	C3—C4—C5—C6	−0.4 (3)
Cu1—N1—C2—C1	19.48 (19)	C4—C5—C6—C7	1.6 (3)
O2—C1—C2—N1	164.25 (17)	C5—C6—C7—C3^i^	178.49 (18)
O1—C1—C2—N1	−18.0 (2)	C5—C6—C7—C7^i^	−1.3 (3)
O5—S1—C3—C4	−116.99 (15)		

Symmetry code: (i) −*x*+1, −*y*+2, −*z*+1.

### Hydrogen-bond geometry (Å, º)

**Table:**

*D*—H···*A*	*D*—H	H···*A*	*D*···*A*	*D*—H···*A*
O1*w*—H11···O2^ii^	0.84	2.02	2.741 (2)	143
O1*w*—H12···O5^iii^	0.84	2.10	2.785 (2)	139
O2*w*—H21···O4^iv^	0.84	2.00	2.773 (2)	152
O2*w*—H22···O4^v^	0.84	2.02	2.823 (2)	158
O3*w*—H31···O2^iii^	0.84	1.91	2.676 (2)	151
O3*w*—H32···O3^v^	0.84	1.93	2.691 (2)	150
N1—H1···O5	0.88	2.53	3.079 (3)	121
N1—H2···O2^iii^	0.88	2.50	3.194 (3)	137

Symmetry codes: (ii) −*x*, *y*−1/2, −*z*+1/2; (iii) −*x*+1, *y*−1/2, −*z*+1/2; (iv) *x*−1, *y*, *z*; (v) −*x*+1, −*y*+1, −*z*+1.

## References

[bb1] Barbour, L. J. (2001). *J. Supramol. Chem.* **1**, 189–191.

[bb2] Cai, J. (2004). *Coord. Chem. Rev.* **248**, 1061–1083.

[bb3] Higashi, T. (1995). *ABSCOR* Rigaku Corporation, Tokyo, Japan.

[bb4] Rigaku (1998). *RAPID-AUTO* Rigaku Corporation, Tokyo, Japan.

[bb5] Rigaku/MSC (2002). *CrystalClear* Rigaku/MSC Inc., The Woodlands, Texas, USA.

[bb6] Sheldrick, G. M. (2008). *Acta Cryst.* A**64**, 112–122. 10.1107/S0108767307043930 18156677

[bb7] Westrip, S. P. (2010). *J. Appl. Cryst.* **43**, 920–925.

